# Association between red blood cell distribution width to albumin ratio and prognosis of patients with sepsis: A retrospective cohort study

**DOI:** 10.3389/fnut.2022.1019502

**Published:** 2022-09-23

**Authors:** Weigan Xu, Jianyang Huo, Guojun Chen, Kangyi Yang, Zuhua Huang, Lina Peng, Jingtao Xu, Jun Jiang

**Affiliations:** ^1^Department of Emergency, First People's Hospital of Foshan, Foshan, China; ^2^The Poison Treatment Centre of Foshan, First People's Hospital of Foshan, Foshan, China

**Keywords:** sepsis, red blood cell distribution width, albumin, RAR, prognosis, MIMIC-IV

## Abstract

**Background:**

Red blood cell distribution width (RDW) to albumin ratio (RAR) is associated with poor prognosis in diabetic comorbidities and cancer. However, the association between RAR and prognosis in patients with sepsis remains unclear, which was investigated in this study.

**Methods:**

We conducted a retrospective cohort study based on the Medical Information Mart for Intensive Care (MIMIC) IV version 2.0 database. The primary outcome of this study was 28-day mortality. Secondary outcomes included 90-day mortality, in-hospital mortality, length of hospital stay, and length of intensive care unit (ICU) stay. Multivariate regression analysis and subgroup analysis were performed to investigate the association between RAR and prognosis in patients with sepsis.

**Results:**

A total of 14,639 participants were included in this study. The mean age of the participants was 65.2 ± 16.3 years and the mean RAR was 5.5 ± 1.9 % /g/dl. For 28-day mortality, after adjusting for covariates, HRs [95% confidence intervals (CIs)] for tertiles 2 (4.4–5.8) and 3 (RAR > 5.8) were 1.33 (1.20, 1.46) and 1.98 (1.79, 2.19), respectively. Similar results were observed for 90-day mortality and in-hospital mortality. According to Kaplan-Meier curve analysis, the higher RAR group had higher 28-day mortality and 90-day mortality.

**Conclusion:**

Our study shows that RAR is significantly associated with poor clinical prognosis in sepsis. The higher the RAR, the higher the 28-day, 90-day, and in-hospital mortality.

## Introduction

Sepsis is a clinical syndrome caused by infection that manifests as systemic inflammation, and the host can suffer from organ dysfunction and even life-threatening effects caused by a dysregulated immune response to infection (1). Although some progress has been made in the management of sepsis, with morbidity and mortality rates trending downward year by year, reports indicate that the incidence of sepsis still ranges from 30 to 80% annually (2) and remains the leading cause of hospital deaths (3). Accurate prediction of the prognosis of sepsis is vital, which will facilitate early aggressive intervention (4). Unfortunately, although some scoring systems have been shown to correlate with outcomes in patients with sepsis (5–8), these scoring systems are inconvenient to use due to the numerous indicators involved, and cannot be used as a satisfactory predictive tool in clinical practice. Therefore, there is an urgent need for convenient biomarkers with good predictive power to help physicians identify high-risk patients and make treatment decisions.

Red blood cell distribution width (RDW) is a common clinical hematology indicator that reflects the heterogeneity of red blood cell size. As a simple and inexpensive parameter, RDW has been successfully used to predict the prognosis of many diseases, including cardiovascular disease, kidney disease, diabetes mellitus, and liver disease (9). Recent studies have also shown that RDW is significantly associated with the mortality of sepsis (10–15). The predictive power of RDW for mortality in sepsis is comparable to that of the Sepsis Associated Organ Failure Assessment (SOFA) and the Acute Physiology and Chronic Health Evaluation-II (APACHE-II) (16). Serum albumin is a negative phase protein that not only reflects systemic nutritional status but also has anti-inflammatory effects by reducing oxidative stress and inhibiting apoptosis of endothelial cells (17, 18). Serum albumin has also been reported to be a biomarker of prognosis in patients with sepsis (19). RDW to albumin ratio (RAR) is a novel and simple biomarker of inflammation. Previous studies have demonstrated that RAR is associated with the prognosis of diabetic ketoacidosis (20), diabetic retinopathy (21), and cancer (22). Previous studies have demonstrated the effectiveness of RAR in predicting the prognosis of diseases such as diabetic ketoacidosis, diabetic retinopathy, and cancer. However, it is unclear whether RAR is associated with the prognosis of patients with sepsis.

In this study, we evaluated the association between RAR and the prognosis of patients with sepsis. We presented the following articles according to the Strengthening the Reporting of Observational Studies in Epidemiology (STROBE) report checklist (23).

## Methods

### Data sources

Data for this retrospective study was extracted from the Medical Information Mart for Intensive Care (MIMIC)-IV version 2.0 database, which contains comprehensive data on 315,460 inpatients from 2008 to 2019. To access the MIMIC-IV version 2.0 database, we completed a training course on the National Institutes of Health (NIH) website and passed the “Protecting Human Research Participants” exam (author certification number: 46450588). The database was approved by the Institutional Review Board of the Massachusetts Institute of Technology and Beth Israel Deaconess Medical Center. To protect patient privacy, all private information in the database depository has been removed. Thus, informed consent and the ethical approval statement were waived for this study. The study was consistent with the Declaration of Helsinki-compliant principles.

### Study population

To screen participants for this study, participants who met the following criteria were included in the study: (1) participants with a diagnosis of sepsis; (2) age ≥18 years; and (3) length of intensive care unit (ICU) stay ≥24 h. Exclusion criteria were as follows: (1) Patients with Sequential Organ Failure Assessment (SOFA) score < 2 were excluded; (2) Patients without RDW or albumin recording were excluded. The diagnosis of sepsis was based on the sepsis 3.0 criteria, which defined sepsis as a suspected or confirmed infection with a SOFA score of 2 or more (1).

### Data extraction

We extracted the following variables from the MIMIC-IV version 2.0 database: sex, age, ethnicity, weight, comorbidities [myocardial infarction, congestive heart failure (CHF), cerebrovascular disease, chronic lung disease, liver disease, diabetes, renal disease, and malignancy], mean blood pressure (MBP), respiratory rate, heart rate, temperature, pulse oxygen saturation (SpO_2_), SOFA score, Simplified Acute Physiology Score (SAPS) II, ventilator use, vasopressors use and renal replacement therapy (RRT) use. Laboratory variables included white blood cells (WBC), hemoglobin (HGB), platelets (PLT), hematocrit (HCT), RDW, albumin, anion gap, sodium, chloride, glucose, blood urea nitrogen (BUN), serum creatinine (Scr), partial thromboplastin time (PPT), alanine transaminase (ALT). Exposure factor RAR is equal to RDW divided by albumin. Data were extracted through Navicat Premium version 15.0. For missing data in continuous variables, we impute with the median of non-missing values. Covariates will be excluded if they have ≥10% missing values.

### Study endpoints

In this study, the primary outcome was 28-day mortality. Secondary outcomes included 90-day mortality, in-hospital mortality, length of hospital stay, and length of ICU stay.

### Statistical analysis

All participants were divided into three groups according to the tertiles of RAR for descriptive analysis. Data for continuous variables were expressed as mean ± standard deviation (SD) or median (IQR), and between-group differences were compared by *t*-test or one-way ANOVA. For categorical variables, data were expressed as frequencies or percentages and analyzed using Chi-square or Fisher test.

Multivariate Cox proportional hazards models for the association between RAR and 28 and 90-day mortality were constructed, and the hazard ratio (HR) of mortality was calculated. We also used logistic regression and linear regression to evaluate the association between RAR and in-hospital mortality, length of ICU stay, and length of hospital stay, respectively. In the multiple regression analysis models, adjusted covariates were selected based on the association of the covariates with clinical outcomes or a change in the effective estimate of more than 10%. In model I, no covariates was adjusted. Model II adjusted for sex and age. In model III, the covariates included sex, age, ethnicity, weight, SAPS II score, Charlson Comorbidity Index, SOFA score, septic shock, myocardial infarction, CHF, cerebrovascular disease, chronic lung disease, liver disease, diabetes, renal disease, malignancy. In model IV, respiratory rate, heart rate, temperature, SpO_2_, MBP, WBC, HGB, PLT, anion gap, sodium, chloride, Scr, BUN, glucose, PTT, ALT, RRT use, ventilator use, and vasopressor use were further adjusted.

We used a smoothed curve fit to assess the association between RAR and sepsis 28-day mortality. Kaplan-Meier curve method was used to compare the probability of survival for different levels of RAR groups. ROC curve analysis was applied to evaluate whether RAR combined with SAPS II score and SOFA score could improve the predictive value of 28-day mortality in sepsis. Statistical analyses and plotting were performed using the R software (version 4.0.0) and free statistics software (version 1.5). A two-sided *p* < 0.05 was considered statistically significant.

### Subgroup analysis and sensitivity analysis

To assess the robustness of the findings, we explored whether the associations differed between different subgroups, including sex, age, septic shock, myocardial infarction, CHF, chronic lung disease, liver disease, diabetes mellitus, renal disease, malignancy, SOFA score, and SAPS II score. We performed two sensitivity analyses for the results. First, sensitivity analysis was performed after removing patients with missing values. Second, considering the infusion of red blood cells or human serum albumin before ICU admission, there may be effects on the exposure factor RAR. Therefore, another sensitivity analysis was performed after removing patients who were transfused with red blood cells and human serum albumin2 days before ICU admission.

## Results

### Population and baseline characteristics

**Figure 1:**
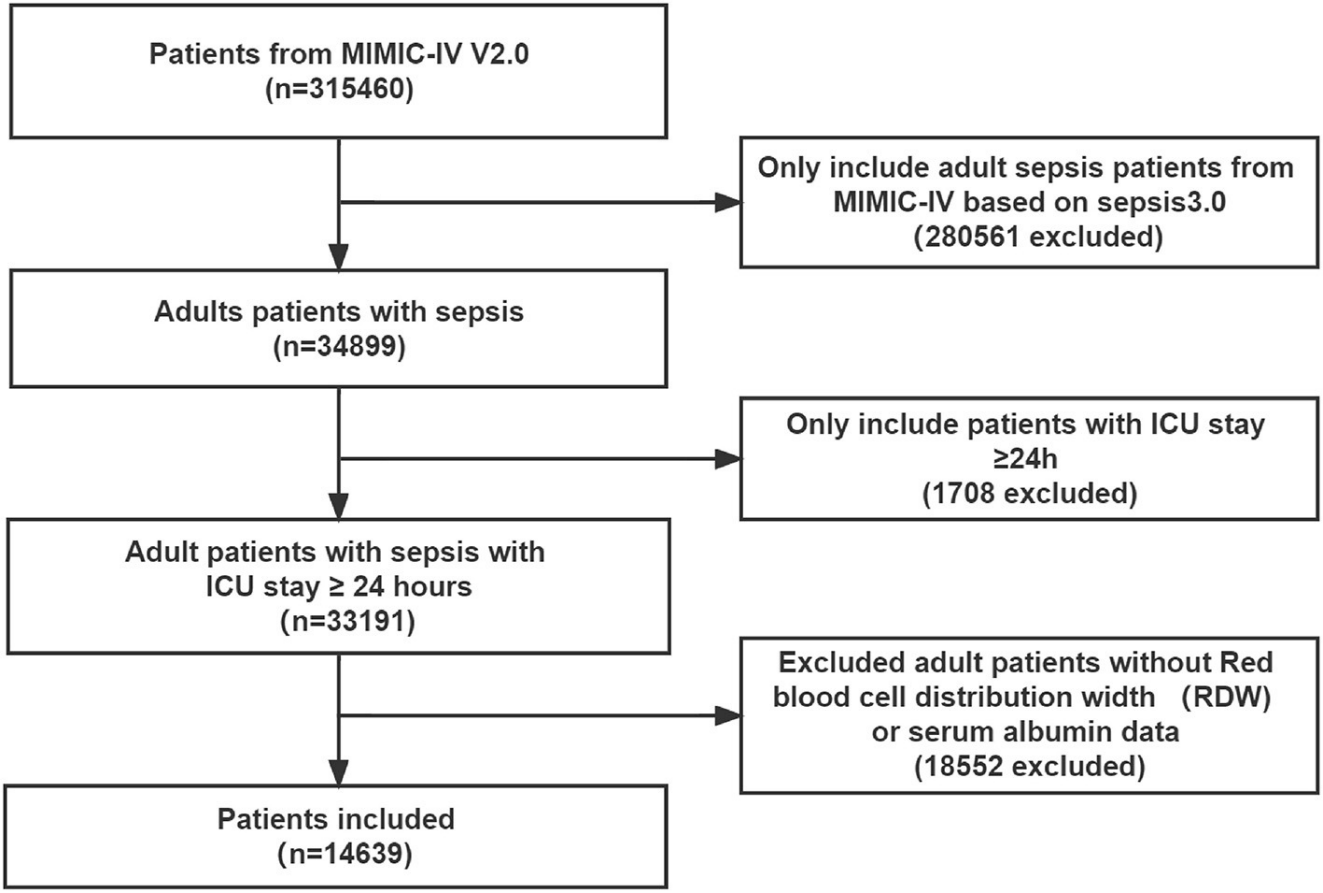
Flowchart of study patient enrollment. ICU, intensive care unit.

Of the 315,460 patients in the MIMIC-IV version 2.0 database, a total of 34,899 met the sepsis-3.0 definition. All patients were ≥18 years old, of which 1708 records were excluded due to length of ICU stay < 24 h, and 18,552 records were excluded due to lack of RDW or albumin information. Therefore, 14,639 patients with sepsis were finally included in this study (Figure 1).

The demographic sample characteristics of all participants are shown in Table 1. The mean age of the patients was 65.2 years, of which 6,261 (42.8%) were female and 8,379 (57.2%) were male. The mean baseline value of RAR was 5.5 ± 1.9 %/g/dl. The majority of patients were white (64.6%). The main comorbidities were CHF (32.9%) and diabetes mellitus (32.7%). Based on RAR values, participants in this study were divided into tertiles (< 4.4, 4.4–5.8, and >5.8). Patients with higher RAR had a higher SOFA score, SAPS II score, and RDW, but lower HGB, HCT, and albumin. Among patients with high RAR, the prevalence of liver disease and malignancy is significantly higher, and the probability of progression to septic shock is also greater. There was a trend toward increased use of vasopressor and RRT in patients with higher RAR. For the study endpoints, the 28, 90-day, and in-hospital mortality were 25.1, 35.6, and 20.8%, respectively. The mean length of hospital stay and ICU stay were 15.0 ± 16.4 days and 6.1 ± 6.8 days, respectively. Patients with elevated RAR had significantly higher 28, 90-day, and in-hospital mortality, and longer length of hospital stay and ICU stay (all *P* < 0.001).

**Table 1:** Baselineclinical and laboratory characteristics of the study patients.

**Characteristic**	**RAR**				***p*-value**
	**Total**	**Tertile 1**	**Tertile 2**	**Tertile 3**	
		** < 4.4**	**4.4-5.8**	**>5.8**	
	**(*n =* 14,639)**	**(*n =* 4,880)**	**(*n =* 4,876)**	**(*n =* 4,883)**	
Sex, *n* (%)					< 0.001
Male	8,378 (57.2)	2,930 (60.0)	2,727 (55.9)	2,721 (55.7)	
Female	6,261 (42.8)	1,950 (40.0)	2,149 (44.1)	2,162 (44.3)	
Age (years)	65.2 ± 16.3	64.1 ± 17.7	66.6 ± 15.9	64.9 ± 15.2	< 0.001
Ethnicity, *n* (%)					< 0.001
White	9,461 (64.6)	3,057 (62.6)	3,238 (66.4)	3,166 (64.8)	
Other	5,178 (35.4)	1,823 (37.4)	1,638 (33.6)	1,717 (35.2)	
Weight (kg)	78.3 (65.8, 93.9)	79.5 (66.7, 94.4)	78.1 (65.8, 94.2)	77.5 (65.0, 92.9)	< 0.001
HR (bpm)	93.9 ± 21.3	90.6 ± 20.6	94.4 ± 21.3	96.7 ± 21.6	< 0.001
RR (bpm)	20.8 ± 6.3	20.4 ± 6.1	20.9 ± 6.3	21.0 ± 6.5	< 0.001
Temperature (°C)	36.8 ± 0.9	36.8 ± 0.9	36.8 ± 1.0	36.7 ± 1.0	< 0.001
SPO_2_ (%)	98.0 (95.0, 100.0)	98.0 (95.0, 100.0)	98.0 (95.0, 100.0)	98.0 (95.0, 100.0)	< 0.001
MBP (mmHg)	82.3 ± 19.9	86.6 ± 19.9	81.8 ± 19.5	78.4 ± 19.3	< 0.001
SASP II score	42.2 ± 14.8	37.9 ± 13.7	42.7 ± 14.4	45.8 ± 15.0	< 0.001
Charlson comorbidity index	6.3 ± 3.0	5.4 ± 2.9	6.6 ± 3.0	6.9 ± 3.0	< 0.001
SOFA score	4.0 ± 2.3	3.4 ± 1.8	4.0 ± 2.2	4.6 ± 2.6	< 0.001
Septic shock, *n* (%)	3,443 (23.5)	700 (14.3)	1,176 (24.1)	1567 (32.1)	< 0.001
Myocardial infarction, *n* (%)	2,571 (17.6)	901 (18.5)	920 (18.9)	750 (15.4)	< 0.001
CHF, *n* (%)	4,817 (32.9)	1,443 (29.6)	1,789 (36.7)	1,585 (32.5)	< 0.001
Cerebrovascular disease, *n* (%)	2,099 (14.3)	973 (19.9)	612 (12.6)	514 (10.5)	< 0.001
Chronic lung disease, *n* (%)	3,933 (26.9)	1,206 (24.7)	1,468 (30.1)	1,259 (25.8)	< 0.001
Liver disease, *n* (%)	3,711 (25.4)	728 (14.9)	1212 (24.9)	1,771 (36.3)	< 0.001
Diabetes, *n* (%)	4,788 (32.7)	1,466 (30.0)	1,731 (35.5)	1,591 (32.6)	< 0.001
Renal disease, *n* (%)	3,941 (26.9)	1,032 (21.1)	1,515 (31.1)	1,394 (28.5)	< 0.001
Malignancy, *n* (%)	2,274 (15.5)	436 (8.9)	756 (15.5)	1,082 (22.2)	< 0.001
WBC ( × 10^9^)	13.5 ± 11.3	12.9 ± 8.3	13.6 ± 13.2	13.8 ± 11.7	< 0.001
HGB (g/dl)	10.7 ± 2.5	12.3 ± 2.2	10.4 ± 2.2	9.3 ± 2.1	< 0.001
PLT ( × 10^12^)	195.0 (125.0, 276.5)	209.0 (155.8, 273.0)	194.0 (123.0, 283.0)	169.0 (94.0, 273.0)	< 0.001
HCT (%)	32.9 ± 7.5	37.3 ± 6.6	32.3 ± 6.9	29.0 ± 6.5	< 0.001
RDW (%)	15.9 ± 2.7	14.0 ± 1.3	15.7 ± 1.9	18.0 ± 3.0	< 0.001
Albumin (g/dL)	3.1 ± 0.7	3.8 ± 0.4	3.1 ± 0.4	2.5 ± 0.5	< 0.001
RAR (%/g/dL)	5.5 ± 1.9	3.7 ± 0.4	5.1 ± 0.4	7.6 ± 1.8	< 0.001
Anion gap (mmol/L)	16.0 (13.0, 20.0)	17.0 (14.0, 20.0)	16.0 (13.0, 20.0)	15.0 (13.0, 19.0)	< 0.001
Sodium (mmol/L)	137.7 ± 6.6	137.8 ± 6.1	137.8 ± 6.7	137.4 ± 6.9	< 0.001
Chloride (mmol/L)	101.9 ± 7.8	101.1 ± 7.1	101.9 ± 8.0	102.9 ± 8.2	< 0.001
Glucose (mg/d)	133.0 (105.0, 178.0)	139.0 (111.0, 186.2)	133.0 (106.0, 180.0)	125.0 (99.0, 168.0)	< 0.001
Scr (mg/dL)	1.2 (0.8, 2.1)	1.1 (0.8, 1.7)	1.3 (0.9, 2.2)	1.3 (0.8, 2.3)	< 0.001
BUN (mmol/L)	26.0 (16.0, 44.0)	21.0 (14.0, 34.0)	28.0 (17.0, 48.0)	30.0 (18.0, 50.0)	< 0.001
PTT (s)	32.0 (27.6, 39.0)	29.9 (26.3, 36.4)	31.9 (27.4, 38.5)	35.0 (29.5, 43.7)	< 0.001
ALT (IU/L)	30.0 (17.0, 80.0)	30.0 (18.0, 83.0)	30.0 (17.0, 80.2)	31.0 (17.0, 77.0)	0.328
RRT use, *n* (%)	1,294 (8.8)	255 (5.2)	436 (8.9)	603 (12.3)	< 0.001
Ventilator use, *n* (%)	7,217 (49.3)	2,449 (50.2)	2,358 (48.4)	2,410 (49.4)	0.018
Vasopressor use, *n* (%)	6,492 (44.3)	1,724 (35.3)	2,180 (44.7)	2,588 (53.0)	< 0.001
28-day mortality, *n* (%)	3,677 (25.1)	787 (16.1)	1,150 (23.6)	1,740 (35.6)	< 0.001
90-day mortality, *n* (%)	4,918 (33.6)	1,038 (21.3)	1,572 (32.2)	2,308 (47.3)	< 0.001
In-hospital mortality (%)	3,048 (20.8)	649 (13.3)	937 (19.2)	1,462 (29.9)	< 0.001
Los hospital (day)	15.0 ± 16.4	12.2 ± 13.0	14.3 ± 14.9	18.6 ± 20.0	< 0.001
Los ICU (day)	6.1 ± 6.8	5.8 ± 6.9	5.9 ± 6.5	6.5 ± 7.0	< 0.001

Data are weighted estimates, and values are presented as means ± standard deviation or means (percentage).

RR, respiratory rate; HR, heart rate; MBP, mean blood pressure; SAPS II, Simplified Acute Physiology Score II; SOFA, Sequential Organ Failure Assessment; CHF, Congestive heart failure; WBC, white blood cell; HCT, hematocrit; HGB, Hemoglobin; PLT, Platelet; RDW, red blood cell distribution width; RAR, red blood cell distribution width to albumin ratio; BUN, blood urea nitrogen; Scr, serum creatinine; PPT, partial thromboplastin time; RRT, renal replacement therapy.

### Association between RAR and clinical outcomes

**Figure 2:**
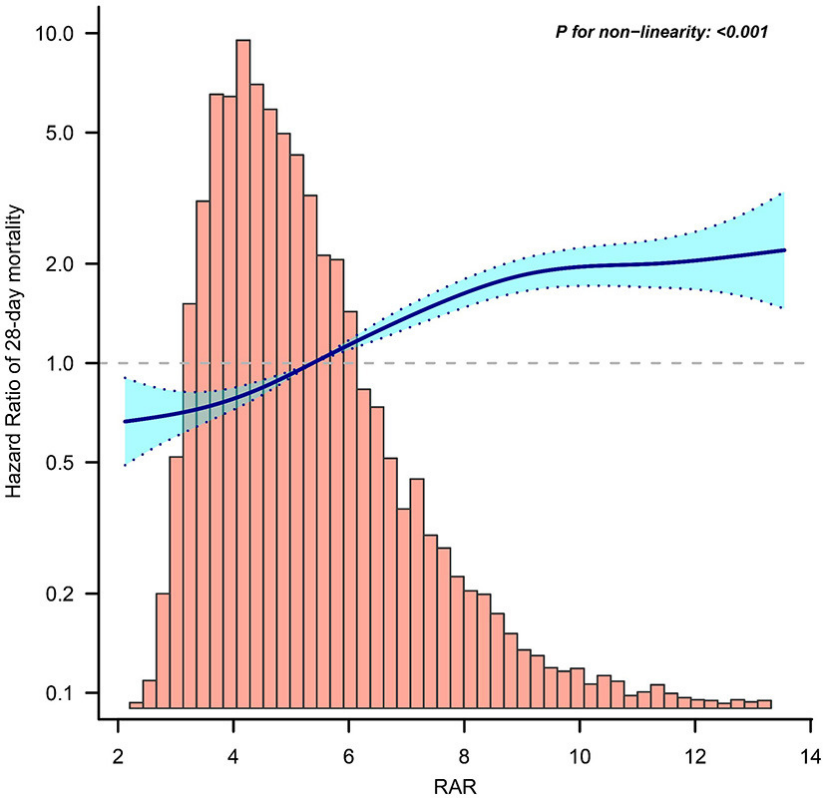
Curve fitting of RAR and 28-day mortality in patients with sepsis. RAR, Red blood cell distribution width to albumin ratio.

To evaluate the linear association between RAR and mortality in patients with sepsis, we performed a smooth curve fitting. After adjusting for confounding variables, a linear association between RAR and 28-day mortality was observed (Figure 2). In addition, a similar linear association was observed in the analysis of 90-day mortality or in-hospital mortality (Supplementary Figures S1, S2).

**Figure 3:**
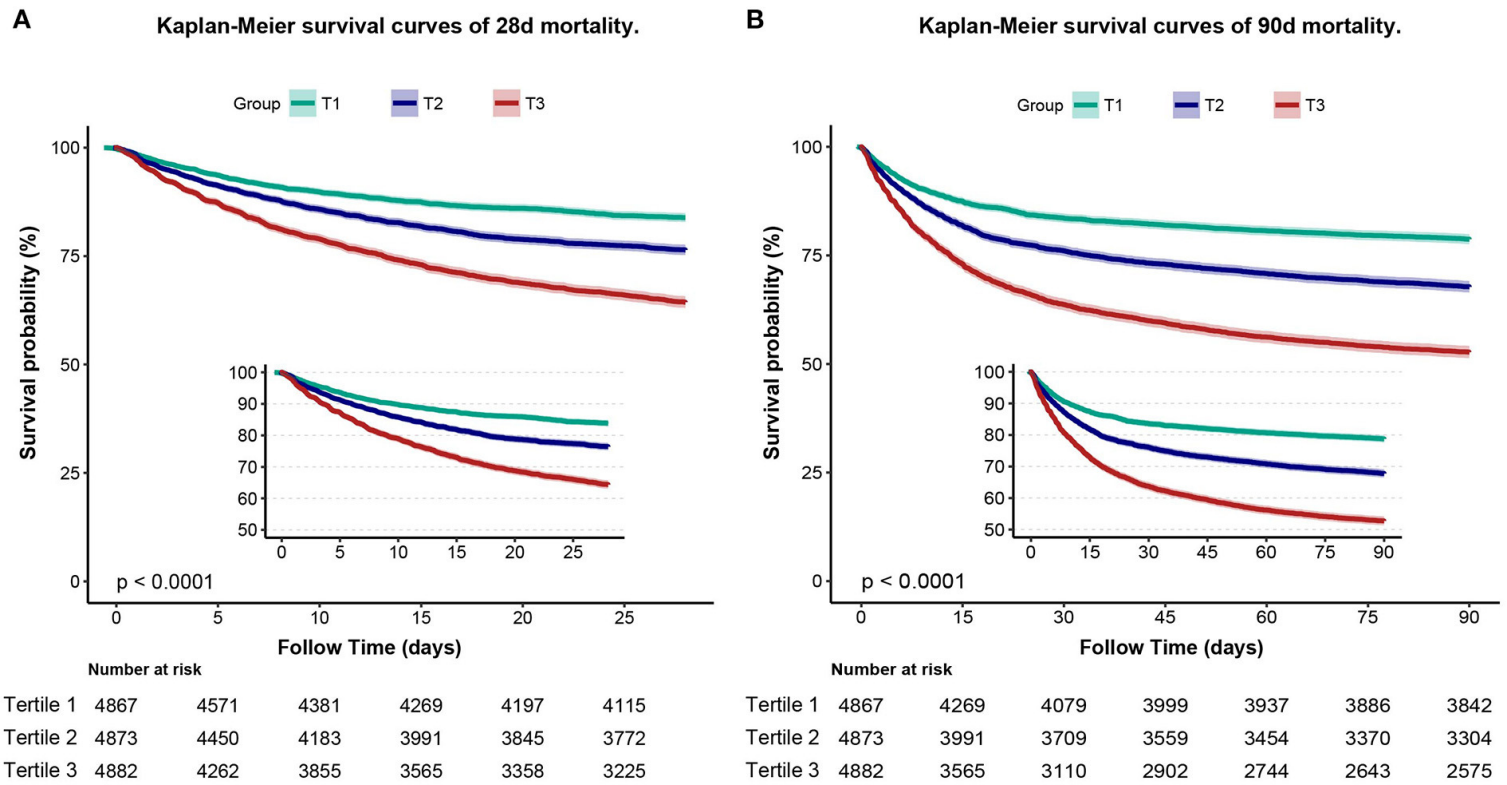
Kaplan–Meier curve of 28-day mortality for patients with sepsis **(A)**. Kaplan–Meier curve of 90-day mortality for patients with sepsis **(B)**.

To assess cumulative survival at different levels of RAR, we generated 28-day survival curves for patients with sepsis by stratifying according to the RAR tertiles. Kaplan-Meier analysis showed that patients in the low RAR group had a significantly higher 28-day survival (*p* < 0.001). In addition, similar results were observed in the 90-day survival curves (Figure 3).

After univariate Cox regression analysis (Supplementary Table S1), extended multivariate models revealed significant associations between RAR and different clinical outcomes (Table 2). For 28-day mortality, in model I unadjusted for variables, the HRs (95% CI) for tertile 2 and tertile 3 were 1.52 (1.39, 1.67) and 2.47 (2.27, 2.69), respectively, compared with the reference group tertile 1 (*p* < 0.001). This association remained statistically significant even after adjusting for sex, age, ethnicity, weight, myocardial infarction, CHF, cerebrovascular disease, chronic lung disease, liver disease, diabetes, kidney disease, malignancy, HR, RR, temperature, SpO_2_, MBP, WBC, HGB, PLT, HCT, anon gap, sodium, chloride, Scr, BUN, glucose, PTT, ALT, RRT use, ventilator use, and vasopressor use. In model IV, the adjusted HR (95% CI) was 1.26 (1.14, 1.39) and 1.74 (1.57, 1.93) for tertile 2 and tertile 3, respectively, compared to the reference group tertile 1 (*p* < 0.001). When analyzed as a continuous variable, RAR was associated with 28-day mortality. HRs (95% CI) in the four models were 1.14 (1.13, 1.15), 1.14 (1.13, 1.15), 1.09 (1.08, 1.11), and 1.09 (1.08, 1.11), respectively (all *p* < 0.001). In the analysis of in-hospital mortality and 90-day mortality, similar results were observed. In addition, we analyzed the association between RAR and length of hospital stay or ICU stay. Linear regression results showed that the beta values (95% CIs) for the length of ICU stay in the four models were 0.15 (0.10, 0.21), 0.16 (0.10, 0.21), 0.08 (0.02, 0.14), and 0.09 (0.03, 0.16), respectively. The beta values (95% CIs) for length of hospital stay in the four models were 1.39 (1.26, 1.53), 1.41 (1.27, 1.54), 1.17 (1.02, 1.31) and 0.78 (0.62, 0.94), respectively (all *p* < 0.05).

**Table 2:** Hazard ratio (HR) [95% confidence intervals (CIs)] for mortality across groups of ratio of red blood cell distribution width (RDW) to albumin (RAR) level.

**Variable**	**Model I**		**Model II**		**Model III**		**Model IV**	
	**HR (95% CI)**	***p*-value**	**HR (95% CI)**	***p*-value**	**HR (95% CI)**	***p*-value**	**HR (95% CI)**	***p*-value**
**Primary outcomes**								
**28-day mortality**								
RAR	1.14 (1.13~1.15)	< 0.001	1.14 (1.13~1.15)	< 0.001	1.09 (1.08~1.11)	< 0.001	1.09 (1.08~1.11)	< 0.001
Tertile								
1st Tertile (< 4.4)	Ref		Ref		Ref		Ref	
2st Tertile (4.4-5.8)	1.52 (1.39~1.67)	< 0.001	1.48 (1.35~1.62)	< 0.001	1.22 (1.11~1.34)	< 0.001	1.26 (1.14~1.39)	< 0.001
3st Tertile (>5.8)	2.47 (2.27~2.69)	< 0.001	2.51 (2.31~2.73)	< 0.001	1.67 (1.53~1.83)	< 0.001	1.74 (1.57~1.93)	< 0.001
p for trend		< 0.001		< 0.001		< 0.001		< 0.001
**Secondary outcomes**								
**90-day mortality**								
RAR	1.14 (1.13~1.15)	< 0.001	1.14 (1.13~1.15)	< 0.001	1.10 (1.09~1.11)	< 0.001	1.10 (1.09~1.11)	< 0.001
Tertile								
1st Tertile (< 4.4)	Ref		Ref		Ref		Ref	
2st Tertile (4.4-5.8)	1.61 (1.49~1.75)	< 0.001	1.57 (1.45~1.70)	< 0.001	1.30 (1.20~1.41)	< 0.001	1.31 (1.20~1.42)	< 0.001
3st Tertile (>5.8)	2.63 (2.45~2.83)	< 0.001	2.69 (2.50~2.89)	< 0.001	1.83 (1.69~1.97)	< 0.001	1.83 (1.68~2.00)	< 0.001
*p* for trend		< 0.001		< 0.001		< 0.001		< 0.001
**In-hospital mortality** ^**a**^								
RAR	1.27 (1.25~1.30)	< 0.001	1.28 (1.26~1.31)	< 0.001	1.18 (1.15~1.20)	< 0.001	1.19 (1.16~1.23)	< 0.001
Tertile								
1st Tertile (< 4.4)	Ref		Ref		Ref		Ref	
2st Tertile (4.4-5.8)	1.55 (1.39~1.73)	< 0.001	1.51 (1.35~1.68)	< 0.001	1.17 (1.04~1.32)	< 0.001	1.21 (1.06~1.37)	< 0.001
3st Tertile (>5.8)	2.79 (2.51~3.09)	< 0.001	2.80 (2.53~3.11)	< 0.001	1.75 (1.56~1.97)	< 0.001	1.80 (1.57~2.07)	< 0.001
p for trend		< 0.001		< 0.001		< 0.001		< 0.001
**Length of ICU stay** ^**b**^	0.15 (0.10~0.21)	< 0.001	0.16 (0.10~0.21)	< 0.001	0.08 (0.02~0.14)	0.011	0.09 (0.03~0.16)	0.007
**Length of hospital stay** ^**b**^	1.39 (1.26~1.53)	< 0.001	1.41 (1.27~1.54)	< 0.001	1.17 (1.02~1.31)	< 0.001	0.78 (0.62~0.94)	< 0.001

Model I adjusted for nothing.

Model II adjusted for sex, age.

Model III adjusted for model II plus weight, ethnicity, SAPS II score, Charlson Comorbidity Index, SOFA score, septic shock, myocardial infarct, CHF, cerebrovascular disease, chronic lung disease, liver disease, diabetes, renal disease, malignancy.

Model IV adjusted for Model III plus HR, RR, temperature, SpO_2_, MBP, WBC, HCT, HGB, PLT, anion gap, sodium, chloride, Scr, BUN, glucose, PTT, ALT, RRT use, ventilator use, vasopressor use.

^a^Logistic regression was used to evaluate the association between RAR and in-hospital mortality. The results were expressed as odds ratio (95% CIs).

^b^Linear regression was used to evaluate the association between RAR and length of stay. The results were expressed as β (95% CIs).

HR, hazard ratio; CI, confidence interval; RR, respiratory rate; HR, heart rate; MBP, mean blood pressure; SAPS II, Simplified Acute Physiology Score II; SOFA, Sequential Organ Failure Assessment; CHF, Congestive heart failure; WBC, white blood cell; HCT, hematocrit; HGB, hemoglobin; PLT, platelet; RAR, red blood cell distribution width to albumin ratio; Scr, serum creatinine; BUN, blood urea nitrogen; PPT, partial thromboplastin time; RRT, renal replacement therapy.

### Receiver operating characteristic analysis

To further evaluate the predictive value of RAR, RAW, albumin, SOFA score, SAPS II score, and RAR combined with different scoring systems in patients with sepsis for 28-day mortality, we constructed receiver operating characteristic (ROC) curves (Supplementary Figure S3; Supplementary Table S2). The results showed that the area under the ROC curve (AUC) (95% CI) for RAR, RDW, albumin, SOFA score, and SAPS II score were 0.633 (0.623, 0.644), 0.614 (0.604, 0.625), 0.602 (0.591, 0.613), 0.603 (0.593, 0.614), and 0.726 (0.717, 0.735), respectively. In addition, the AUC (95% CI) for RAR combined with SOFA score or SAPS II score was 0.656 (0.646, 0.667), 0.743 (0.733, 0.752).

### Subgroup analysis and sensitivity analysis

Table 3 shows the results of the subgroup analysis. There was an interaction between age and RAR on 28-day mortality (p for interaction =0.043). For elderly patients (≥60 years), 28-day mortality was higher with increasing RAR. The HRs (95% CI) for tertile 2 and tertile 3 were 1.33 (1.19, 1.49) and 2.10 (1.86, 2.36), respectively, compared with tertile 1 (*p* < 0.001). No significant interactions were observed in other subgroups (*p* for interaction > 0.05).

**Table 3:** Subgroup analysis of the associations between 28-day mortality and the RAR level.

	**No. of patients**	**RAR**			***P* for interaction**
		** < 4.4**	**4.4-5.8**	**>5.8**	
**Sex**					0.851
Male	8,378	1.0	1.27 (1.12~1.44)	1.75 (1.53~2.01)	
Female	6,261	1.0	1.26 (1.08~1.46)	1.77 (1.51~2.07)	
**Age**					0.043
< 60	5,152	1.0	1.04 (0.85~1.26)	1.40 (1.15~1.72)	
≥60	9,487	1.0	1.33 (1.19~1.49)	2.10 (1.86~2.36)	
**Septic shock**					0.113
Yes	3,443	1.0	1.27 (1.14~1.43)	1.69 (1.50~1.92)	
No	11,196	1.0	1.28 (1.05~1.54)	1.91 (1.58~2.32)	
**Myocardial infarction**					0.165
Yes	2,571	1.0	1.24 (1.11~1.39)	1.78 (1.59~2.00)	
No	12,068	1.0	1.35 (1.10~1.65)	1.69 (1.35~2.12)	
**CHF**					0.315
Yes	4,817	1.0	1.22 (1.08~1.38)	1.61 (1.41~1.83)	
No	9,822	1.0	1.33 (1.13~1.56)	1.97 (1.66~2.33)	
**Chronic lung disease**					0.194
Yes	3,933	1.0	1.22 (1.09~1.37)	1.74 (1.54~1.96)	
No	10,706	1.0	1.36 (1.14~1.63)	1.78 (1.47~2.16)	
**Liver disease**					0.683
Yes	3,711	1.0	1.30 (1.17~1.46)	1.79 (1.58~2.02)	
No	10,928	1.0	1.17 (0.95~1.44)	1.62 (1.32~1.98)	
**Diabetes**					0.792
Yes	4,788	1.0	1.27 (1.13~1.43)	1.72 (1.52~1.94)	
No	9,851	1.0	1.26 (1.06~1.50)	1.86 (1.54~2.23)	
**Renal disease**					0.426
Yes	3,941	1.0	1.20 (1.07~1.35)	1.65 (1.46~1.87)	
No	10,698	1.0	1.39 (1.16~1.66)	2.01 (1.66~2.44)	
**Malignancy**					0.052
Yes	2,274	1.0	1.17 (1.05~1.31)	1.66 (1.48~1.86)	
No	12,365	1.0	1.54 (1.20~1.97)	1.88 (1.47~2.41)	
**SOFA score**					0.372
< 4	77,35	1.0	1.39 (1.21~1.61)	1.92 (1.64~2.25)	
≥4	6,904	1.0	1.15 (1.01~1.31)	1.62 (1.41~1.86)	
**SAPS II score**					0.216
< 42	7,683	1.0	1.15 (0.97~1.37)	1.75 (1.45~2.11)	
≥42	6,956	1.0	1.27 (1.13~1.43)	1.71 (1.51~1.93)	

HRs (95% CIs) were derived from Cox proportional hazards regression models. Covariates were adjusted as in model IV (Table 2).

CHF, Congestive heart failure; SOFA, Sequential Organ Failure Assessment; SAPS II, Simplified Acute Physiology Score II.

Two sensitivity analyses were performed to assess the robustness of the study results. After removal of patients with missing values, sensitivity analyses showed that the association between RAR and clinical outcomes in patients with sepsis remained strong (Supplementary Table S3). In addition, after removing patients who had received red blood cells and human serum albumin infusion 2 days before ICU admission, sensitivity analyses were performed again, and the results were consistent with our main finding that RAR remained significantly associated with clinical outcomes in patients with sepsis (Supplementary Table S4).

## Discussion

In this study, we investigate for the first time the association between RAR and poor clinical outcomes in patients with sepsis. We found that in patients with sepsis, elevated RAR was significantly associated with 28 days, 90 days, in-hospital mortality, length of ICU stay, and length of hospital stay. ROC curves showed that RAR had good predictive power for 28-day mortality in patients with sepsis.

Hematopoietic dysfunction occurs frequently during sepsis. Systemic infections and inflammation can inhibit erythropoietin production and affect erythrocyte maturation, leading to an increased proportion of immature erythrocytes in the circulation (24). Inflammatory factors can reduce iron utilization and promote erythrocyte apoptosis, leading to the development of sepsis-associated anemia (25, 26). In addition, inflammatory factors can also affect the cell membrane glycoproteins and ion channels of erythrocytes, resulting in altered erythrocyte morphology (27, 28). All of these pathological changes increase the heterogeneity of red blood cell volume and lead to elevated RDW. A recent study reported that all-cause mortality in patients with sepsis increased with increasing RDW values and RDW was an effective predictor of sepsis prognosis (15). Serum albumin is an important protein with inflammatory, nutritional, and blood rheological properties, inhibiting platelet activation and aggregation (29, 30). Reduced serum albumin levels are usually associated with increased blood viscosity and impaired endothelial function (31). Albumin has been proposed as a reliable predictor of prognosis in critically ill patients (32). Recent studies have shown that hypoalbuminemia is associated with poor prognosis in patients with sepsis (33, 34).

RAR is a new indicator combining RDW and albumin, which is widely studied in various inflammation-related diseases. Long et al. revealed that RAR is a risk factor for prognosis in patients with aortic aneurysms (35). Zhou et al. reported that high RAR was significantly associated with increased all-cause mortality in diabetic ketoacidosis and an increased incidence of DKA-related infections (20). In addition, RAR showed good predictive power for the prognosis of cancer (22) and acute respiratory distress syndrome (36), both of which are associated with sepsis (37). However, no studies have reported an association between RAR and sepsis. Therefore, we hypothesized that RAR is also associated with prognosis in sepsis. This study enrolled 14,639 patients with sepsis in the intensive care unit. The results showed that RAR was associated with increased patient mortality at 28, 90 days and in-hospital mortality, longer length of stay and ICU stay. It is suggested that the higher the RAR the poorer the clinical prognosis of patients with sepsis.

It is worth mentioning that SOFA score, SAPS II score are clinically used disease severity scores which are associated with prognosis of patients with sepsis. In the present study, we observed that patients with a higher RAR had a higher SOFA score, SAPS II score, a greater probability of progression to septic shock, and a higher proportion of vasopressors and RRT use. Superficially, RAR organically combines RDW and albumin, and reflects the levels of the two factors. In terms of clinical significance, RAR can comprehensively reflect the two pathological states of hematopoietic dysfunction and hypoalbuminemia. The results of the study suggest that there may be an association between RAR and the severity of the disease in patients with sepsis in ICU. In our further subgroup analysis, age (≥60 years) showed an interaction with RARin 28-day mortality, suggesting that RAR may be an important biomarker for predicting the prognosis of elderly patients with sepsis. We are cautious about the findings of subgroup analyses since these results may be influenced by heterogeneity among different populations. These results require further research to be confirmed.

In the present study, we observed that the area under the ROC curve of RAR was superior to that of RDW or albumin alone, suggesting that RAR has a higher predictive value for 28-d morbidity and mortality in sepsis patients than RDW or albumin alone. This can be explained as follows. Although both RDW and albumin are associated with sepsis prognosis and reflect a systemic inflammatory response, they show opposite responses in terms of inflammation. A strong inflammatory response can lead to a significant increase in RAR. In comparison to RDW and albumin alone, The RAR reflects not only the hematopoietic and nutritional status of the patient but also the severity of inflammation. Weng et al. reported that RAR was superior to RDW or albumin alone in predicting prognosis in patients after percutaneous coronary intervention (38), which is similar to the result of this study. In addition, we observed that the area under the ROC curve increased after RAR was combined with SAPS II score or SOFA score, suggesting that RAR combined with SAPS II or SOFA score further improves the predictive power of septic mortality.

Our study had some limitations. First, owing to the retrospective design, the population was a heterogeneous mixture of infection etiologies, although we tried our best to adjust for potential confounding confounders and perform subgroup analyses, it was still difficult to avoid selection bias and confounding bias, which is a limitation of all retrospective studies. Second, some variables in the MIMIC-IV database had a high number of missing values, including C-reactive protein, N-terminal pro-B-type natriuretic peptide (NT-proBNP), troponin t, lactate, and blood gas analysis. Considering that too many missing values may have an impact on the study results, we ultimately did not include these covariates in the regression analysis. Third, we only measured RAR at ICU admission, which may have influenced the results if the patient had been transfused with human serum albumin before ICU admission. Therefore, we performed a sensitivity analysis after excluding this cohort. Fourth, some comorbidities, such as chronic liver disease and chronic kidney disease, may have an impact on RDW and albumin levels. We performed subgroup analyses on these populations to verify the robustness of the results. Finally, our results suggested that RAR is associated with poor clinical prognosis of sepsis. These findings are hypothesis-generating and should be considered exploratory. We believe that a carefully designed, multicenter prospective study is needed to validate our results.

## Conclusions

RAR is a potential prognostic indicator for patients with sepsis and is associated with a poor clinical prognosis. The higher the RAR, the higher the 28, 90-day, and in-hospital mortality.

## Data availability statement

Publicly available datasets were analyzed in this study. This data can be found here: https://physionet.org/content/mimiciv.

## Ethics statement

The studies involving human participants were reviewed and approved by Institutional Review Board of the Massachusetts Institute of Technology and Beth Israel Deaconess Medical Center. Written informed consent for participation was not required for this study in accordance with the National Legislation and the Institutional requirements.

## Author contributions

WX and JJ designed the study. JX, GC, and JH conducted data collection and data analysis. WX wrote the manuscript. KY, ZH, and LP analyzed and interpreted the result. All authors contributed to the article and approved the submitted version.

## Conflict of interest

The authors declare that the research was conducted in the absence of any commercial or financial relationships that could be construed as a potential conflict of interest.

## Publisher's note

All claims expressed in this article are solely those of the authors and do not necessarily represent those of their affiliated organizations, or those of the publisher, the editors and the reviewers. Any product that may be evaluated in this article, or claim that may be made by its manufacturer, is not guaranteed or endorsed by the publisher.

## Abbreviations

RR: respiratory rate
HR: heart rate
MBP: mean blood pressure
SAPS II: Simplified Acute Physiology Score II
SOFA: Sequential Organ Failure Assessment
CHF: Congestive heart failure
WBC: white blood cell
HCT: hematocrit
HGB: Hemoglobin
PLT: Platelet
RDW: red blood cell distribution width
RAR: red blood cell distribution width to albumin ratio
BUN: blood urea nitrogen
Scr: serum creatinine
PPT: partial thromboplastin time
RRT: renal replacement therapy.
