# Supplementary material for: Association between red blood cell distribution width to albumin ratio and prognosis of patients with sepsis: A retrospective cohort study
Source: Front Nutr. 2022 Sep 23;9:1019502. doi: 10.3389/fnut.2022.1019502 (PMC9539557; doi:10.3389/fnut.2022.1019502)
Supplement: Supplementary file 1 [file Data_Sheet_1.docx]

Supplementary Material

| Table S1 Results of univariate analysis of 28-day mortality. | | | | | | | | |  |
| --- | --- | --- | --- | --- | --- | --- | --- | --- | --- |
| **Characteristic** | | | **HR (95% CI)** | | | ***P-value*** | | |  |
| Sex | | | 1.02 (0.96,1.08) | | | 0.523 | |  |  |
| Age | | | 1.02 (1.02,1.02) | | | < 0.001 | |  |  |
| Ethnicity | | | 0.81 (0.76,0.86) | | | < 0.001 | |  |  |
| Weight | | | 1.00 (1.00,1.00) | | | < 0.001 | |  |  |
| HR | | | 1.01 (1.00,1.01) | | | < 0.001 | |  |  |
| RR | | | 1.03 (1.02,1.03) | | | < 0.001 | |  |  |
| Temperature | | | 0.74 (0.73,0.76) | | | < 0.001 | |  |  |
| SpO_2_ | | | 0.97 (0.96,0.97) | | | < 0.001 | |  |  |
| MBP | | | 0.99 (0.99,0.99) | | | < 0.001 | |  |  |
| SAPS II score | | | 1.05 (1.05,1.05) | | | < 0.001 | |  |  |
| Charlson Comorbidity Index | | | 1.13 (1.12,1.14) | | | < 0.001 | |  |  |
| SOFA score | | | 1.15 (1.14,1.17) | | | < 0.001 | |  |  |
| Septic shock | | | 1.96 (1.84,2.09) | | | < 0.001 | |  |  |
| Myocardial infarction | | | 1.28 (1.19,1.38) | | | < 0.001 | |  |  |
| CHF | | | 1.19 (1.11,1.26) | | | < 0.001 | |  |  |
| Cerebrovascular disease | | | 1.34 (1.24,1.45) | | | < 0.001 | |  |  |
| Chronic lung disease | | | 1.04 (0.97,1.12) | | | 0.227 | |  |  |
| Liver disease | | | 1.33 (1.24,1.42) | | | < 0.001 | |  |  |
| Diabetes mellitus | | | 0.93 (0.87,0.99) | | | 0.020 | |  |  |
| Renal disease | | | 1.20 (1.12,1.28) | | | < 0.001 | |  |  |
| Malignancy | | | 1.68 (1.56,1.80) | | | < 0.001 | |  |  |
| WBC | | | 1.01(1.01,1.01) | | | < 0.001 | |  |  |
| HGB | | | 0.95 (0.94,0.96) | | | < 0.001 | |  |  |
| PLT | | | 1.00 (1.00,1.00) | | | < 0.001 | |  |  |
| HCT | | | 0.99 (0.99,0.99) | | | < 0.001 | |  |  |
| RDW | | | 1.11 (1.10,1.12) | | | < 0.001 | |  |  |
| Albumin | | | 0.59 (0.57,0.62) | | | < 0.001 | |  |  |
| RAR | | | 1.14 (1.14,1.15) | | | < 0.001 | |  |  |
| Anion gap | | | 1.05 (1.04,1.05) | | | < 0.001 | |  |  |
| Sodium | | | 1.00 (0.99,1.01) | | | 0.319 | |  |  |
| Chloride | | | 0.99 (0.99,1.00) | | | 0.010 | |  |  |
| Glucose | | | 1.00 (0.99,1.00) | | | 0.797 | |  |  |
| Scr | | | 1.05 (1.03,1.06) | | | < 0.001 | |  |  |
| BUN | | | 1.01 (1.01,1.01) | | | < 0.001 | |  |  |
| PTT | | | 1.01 (1.01,1.01) | | | < 0.001 | |  |  |
| ALT | | | 1.00 (1.00,1.00) | | | < 0.001 | |  |  |
| RRT use | | | 1.64 (1.49,1.80) | | | < 0.001 | |  |  |
| Ventilator use | | | 1.64 (1.54,1.74) | | | < 0.001 | |  |  |
| Vasopressor use | | | 2.04 (1.92,2.17) | | | < 0.001 | |  |  |
| Abbreviations: RR, respiratory rate; HR, heart rate; MBP, mean blood pressure; SpO_2_, pulse oxygen saturation; SAPS II, Simplified Acute Physiology Score II; SOFA, Sequential Organ Failure Assessment; CHF, Congestive heart failure; WBC, white blood cell; HCT, hematocrit; HGB, Hemoglobin; PLT, Platelet; RDW, red blood cell distribution width; RAR, red blood cell distribution width to albumin ratio; BUN, blood urea nitrogen; Scr, serum creatinine; PPT, partial thromboplastin time; RRT, renal replacement therapy. | | | | | | | |  |  |
| Table S2 ROC curve analysis of RAR, RDW, Albumin, SAPS II score, SOFA score, RAR + SOFA score, and RAR +SAPS II score. | | | | | | | | | |
| **index** | **AUC** | **95% CI** | | **sensitivity** | **specificity** | | **cutoff value** | | |
| RAR | 0.633 | 0.623-0.644 | | 51.9 | 67.3 | | 5.62 | | |
| RDW | 0.614 | 0.604-0.625 | | 50.3 | 66.8 | | 16.15 | | |
| Albumin | 0.602 | 0.591-0.613 | | 42.2 | 72.9 | | 2.75 | | |
| SOFA score | 0.603 | 0.593-0.614 | | 59.0 | 56.8 | | 3.50 | | |
| SAPS II score | 0.726 | 0.717-0.735 | | 66.7 | 66.6 | | 43.50 | | |
| RAR + SOFA score | 0.656 | 0.646-0.667 | | 53.4 | 69.9 | | 0.26 | | |
| RAR +SAPS II score | 0.743 | 0.733-0.752 | | 66.7 | 69.8 | | 0.25 | | |
| RDW, red blood cell distribution width; RAR, red blood cell distribution width to albumin ratio; SOFA, Sequential Organ Failure Assessment; SAPS II, Simplified Acute Physiology Score II. | | | | | | | | | |

| Table S3 Sensitivity analysis after removing patients with missing values | | | | | | | | | | | |
| --- | --- | --- | --- | --- | --- | --- | --- | --- | --- | --- | --- |
| Variable | Model I | |  | Model II | |  | Model III | |  | Model IV | |
|  | HR (95% CI) | *P value* |  | HR (95% CI) | *P value* |  | HR (95% CI) | *P value* |  | HR (95% CI) | *P value* |
| Primary outcomes |  |  |  |  |  |  |  |  |  |  |  |
| 28-day mortality |  |  |  |  |  |  |  |  |  |  |  |
| RAR | 1.13 (1.12~1.14) | <0.001 |  | 1.14 (1.13~1.15) | <0.001 |  | 1.09 (1.08~1.10) | <0.001 |  | 1.09 (1.08~1.10) | <0.001 |
| Tertile | |  |  |  |  |  |  |  |  |  |  |
| 1st Tertile (<4.4) | Ref |  |  | Ref |  |  | Ref |  |  | Ref |  |
| 2st Tertile (4.4-5.8) | 1.49 (1.36~1.64) | <0.001 |  | 1.46 (1.33~1.60) | <0.001 |  | 1.21 (1.10~1.33) | <0.001 |  | 1.28 (1.15~1.41) | <0.001 |
| 3st Tertile (>5.8) | 2.39 (2.19~2.61) | <0.001 |  | 2.44 (2.24~2.67) | <0.001 |  | 1.63 (1.48~1.79) | <0.001 |  | 1.75 (1.57~1.95) | <0.001 |
| p for trend |  | <0.001 |  |  | <0.001 |  |  | <0.001 |  |  | <0.001 |
| Secondary outcomes |  |  |  |  |  |  |  |  |  |  |  |
| 90-day mortality |  |  |  |  |  |  |  |  |  |  |  |
| RAR | 1.13 (1.13~1.14) | <0.001 |  | 1.14 (1.13~1.15) | <0.001 |  | 1.10 (1.09~1.11) | <0.001 |  | 1.09 (1.08~1.10) | <0.001 |
| Tertile |  |  |  |  |  |  |  |  |  |  |  |
| 1st Tertile (<4.4) | Ref |  |  | Ref |  |  | Ref |  |  | Ref |  |
| 2st Tertile (4.4-5.8) | 1.60 (1.48~1.74) | <0.001 |  | 1.57 (1.44~1.70) | <0.001 |  | 1.30 (1.19~1.41) | <0.001 |  | 1.35 (1.23~1.47) | <0.001 |
| 3st Tertile (>5.8) | 2.57 (2.38~2.78) | <0.001 |  | 2.65 (2.45~2.86) | <0.001 |  | 1.80 (1.66~1.95) | <0.001 |  | 1.87 (1.70~2.05) | <0.001 |
| p for trend |  | <0.001 |  |  | <0.001 |  |  | <0.001 |  |  | <0.001 |
| In-hospital mortality^a^ |  |  |  |  |  |  |  |  |  |  |  |
| RAR | 1.26 (1.24~1.29) | <0.001 |  | 1.27 (1.24~1.30) | <0.001 |  | 1.17 (1.14~1.20) | <0.001 |  | 1.18 (1.15~1.22) | <0.001 |
| Tertile |  |  |  |  |  |  |  |  |  |  |  |
| 1st Tertile (<4.4) | Ref |  |  | Ref |  |  | Ref |  |  | Ref |  |
| 2st Tertile (4.4-5.8) | 1.52 (1.36~1.70) | <0.001 |  | 1.48 (1.32~1.66) | <0.001 |  | 1.16 (1.02~1.31) | <0.001 |  | 1.22 (1.06~1.40) | <0.001 |
| 3st Tertile (>5.8) | 2.67 (2.40~2.98) | <0.001 |  | 2.71 (2.43~3.02) | <0.001 |  | 1.70 (1.50~1.92) | <0.001 |  | 1.77 (1.53~2.05) | <0.001 |
| p for trend |  | <0.001 |  |  | <0.001 |  |  | <0.001 |  |  | <0.001 |
| Length of ICU stay^b^ | 0.16 (0.10~0.22) | <0.001 |  | 0.16 (0.10~0.22) | <0.001 |  | 0.08 (0.02~0.15) | 0.008 |  | 0.11 (0.04~0.17) | 0.003 |
| Length of hospital stay^b^ | 1.36 (1.22~1.50) | <0.001 |  | 1.37 (1.23~1.51) | <0.001 |  | 1.13 (0.98~1.28) | <0.001 |  | 0.81 (0.64~0.98) | <0.001 |
| *Note*:  Model I adjusted for nothing.  Model II adjusted for sex, age.  Model III adjusted for model II plus weight, ethnicity, SAPS II score, Charlson Comorbidity Index, SOFA score, septic shock, myocardial infarct, CHF, cerebrovascular disease, chronic lung disease, liver disease, diabetes mellitus, renal disease, malignancy.  Model IV adjusted for Model III plus HR, RR, temperature, SpO_2_, MBP, WBC, HCT, HGB, PLT, anion gap, sodium, chloride, Scr, BUN, glucose, PTT, ALT, RRT use, ventilator use, vasopressor use.  ^a^ Logistic regression was used to evaluate the association between RAR and in-hospital mortality. The results were expressed as odds ratio (95% CIs).  ^b^ Linear regression was used to evaluate the association between RAR and length of stay. The results were expressed as β (95% CIs).  Abbreviations: HR, hazard ratio; CI, confidence interval; RR, respiratory rate; HR, heart rate; MBP, mean blood pressure; SpO_2_, pulse oxygen saturation; SAPS II, Simplified Acute Physiology Score II; SOFA, Sequential Organ Failure Assessment; CHF, Congestive heart failure; WBC, white blood cell; HCT, hematocrit; HGB, hemoglobin; PLT, platelet; RAR, red blood cell distribution width to albumin ratio; Scr, serum creatinine; BUN, blood urea nitrogen; PPT, partial thromboplastin time; RRT, renal replacement therapy | | | | | | | | | | | |

| Table S4 Sensitivity analysis after removing patients who had received red blood cells and human serum albumin infusion 2 days before ICU admission | | | | | | | | | | | |
| --- | --- | --- | --- | --- | --- | --- | --- | --- | --- | --- | --- |
| Variable | Model I | |  | Model II | |  | Model III | |  | Model IV | |
|  | HR (95% CI) | *P value* |  | HR (95% CI) | *P value* |  | HR (95% CI) | *P value* |  | HR (95% CI) | *P value* |
| Primary outcomes |  |  |  |  |  |  |  |  |  |  |  |
| 28-day mortality |  |  |  |  |  |  |  |  |  |  |  |
| RAR | 1.14 (1.13~1.15) | <0.001 |  | 1.14 (1.13~1.15) | <0.001 |  | 1.09 (1.08~1.11) | <0.001 |  | 1.10 (1.08~1.11) | <0.001 |
| Tertile | |  |  |  |  |  |  |  |  |  |  |
| 1st Tertile (<4.4) | Ref |  |  | Ref |  |  | Ref |  |  | Ref |  |
| 2st Tertile (4.4-5.8) | 1.52 (1.39~1.67) | <0.001 |  | 1.47 (1.34~1.62) | <0.001 |  | 1.23 (1.12~1.35) | <0.001 |  | 1.29 (1.17~1.42) | <0.001 |
| 3st Tertile (>5.8) | 2.50 (2.3~2.73) | <0.001 |  | 2.53 (2.32~2.76) | <0.001 |  | 1.70 (1.55~1.86) | <0.001 |  | 1.83 (1.65~2.04) | <0.001 |
| p for trend |  | <0.001 |  |  | <0.001 |  |  | <0.001 |  |  | <0.001 |
| Secondary outcomes |  |  |  |  |  |  |  |  |  |  |  |
| 90-day mortality |  |  |  |  |  |  |  |  |  |  |  |
| RAR | 1.14 (1.13~1.15) | <0.001 |  | 1.14 (1.14~1.15) | <0.001 |  | 1.10 (1.09~1.11) | <0.001 |  | 1.10 (1.09~1.11) | <0.001 |
| Tertile |  |  |  |  |  |  |  |  |  |  |  |
| 1st Tertile (<4.4) | Ref |  |  | Ref |  |  | Ref |  |  | Ref |  |
| 2st Tertile (4.4-5.8) | 1.62 (1.50~1.76) | <0.001 |  | 1.57 (1.45~1.71) | <0.001 |  | 1.32 (1.21~1.43) | <0.001 |  | 1.34 (1.23~1.46) | <0.001 |
| 3st Tertile (>5.8) | 2.69 (2.49~2.90) | <0.001 |  | 2.74 (2.54~2.95) | <0.001 |  | 1.87 (1.73~2.03) | <0.001 |  | 1.93 (1.76~2.11) | <0.001 |
| p for trend |  | <0.001 |  |  | <0.001 |  |  | <0.001 |  |  | <0.001 |
| In-hospital mortality^a^ |  |  |  |  |  |  |  |  |  |  |  |
| RAR | 1.28 (1.25~1.30) | <0.001 |  | 1.29 (1.26~1.32) | <0.001 |  | 1.19 (1.16~1.21) | <0.001 |  | 1.22 (1.18~1.25) | <0.001 |
| Tertile |  |  |  |  |  |  |  |  |  |  |  |
| 1st Tertile (<4.4) | Ref |  |  | Ref |  |  | Ref |  |  | Ref |  |
| 2st Tertile (4.4-5.8) | 1.56 (1.40~1.75) | <0.001 |  | 1.51 (1.35~1.69) | <0.001 |  | 1.20 (1.06~1.36) | <0.001 |  | 1.26 (1.11~1.44) | <0.001 |
| 3st Tertile (>5.8) | 2.84 (2.55~3.15) | <0.001 |  | 2.85 (2.56~3.17) | <0.001 |  | 1.82 (1.61~2.06) | <0.001 |  | 1.96 (1.70~2.26) | <0.001 |
| p for trend |  | <0.001 |  |  | <0.001 |  |  | <0.001 |  |  | <0.001 |
| Length of ICU stay^b^ | 0.15 (0.09~0.21) | <0.001 |  | 0.16 (0.10~0.21) | <0.001 |  | 0.09 (0.03~0.15) | 0.006 |  | 0.11 (0.05~0.18) | 0.001 |
| Length of hospital stay^b^ | 1.32 (1.19~1.45) | <0.001 |  | 1.34 (1.21~1.47) | <0.001 |  | 1.17 (1.03~1.32) | <0.001 |  | 0.85 (0.69~1.01) | <0.001 |
| *Note*:  Model I adjusted for nothing.  Model II adjusted for sex, age.  Model III adjusted for model II plus weight, ethnicity, SAPS II score, Charlson Comorbidity Index, SOFA score, septic shock, myocardial infarct, CHF, cerebrovascular disease, chronic lung disease, liver disease, diabetes mellitus, renal disease, malignancy.  Model IV adjusted for Model III plus HR, RR, temperature, SpO_2_, MBP, WBC, HCT, HGB, PLT, anion gap, sodium, chloride, Scr, BUN, glucose, PTT, ALT, RRT use, ventilator use, vasopressor use.  ^a^ Logistic regression was used to evaluate the association between RAR and in-hospital mortality. The results were expressed as odds ratio (95% CIs).  ^b^ Linear regression was used to evaluate the association between RAR and length of stay. The results were expressed as β (95% CIs).  Abbreviations: HR, hazard ratio; CI, confidence interval; RR, respiratory rate; HR, heart rate; MBP, mean blood pressure; SpO_2_, pulse oxygen saturation; SAPS II, Simplified Acute Physiology Score II; SOFA, Sequential Organ Failure Assessment; CHF, Congestive heart failure; WBC, white blood cell; HCT, hematocrit; HGB, hemoglobin; PLT, platelet; RAR, red blood cell distribution width to albumin ratio; Scr, serum creatinine; BUN, blood urea nitrogen; PPT, partial thromboplastin time; RRT, renal replacement therapy | | | | | | | | | | | |
