# Supplementary figures and images for: Association between red blood cell distribution width to albumin ratio and prognosis of patients with sepsis: A retrospective cohort study
Source: Front Nutr. 2022 Sep 23;9:1019502. doi: 10.3389/fnut.2022.1019502 (PMC9539557; doi:10.3389/fnut.2022.1019502)

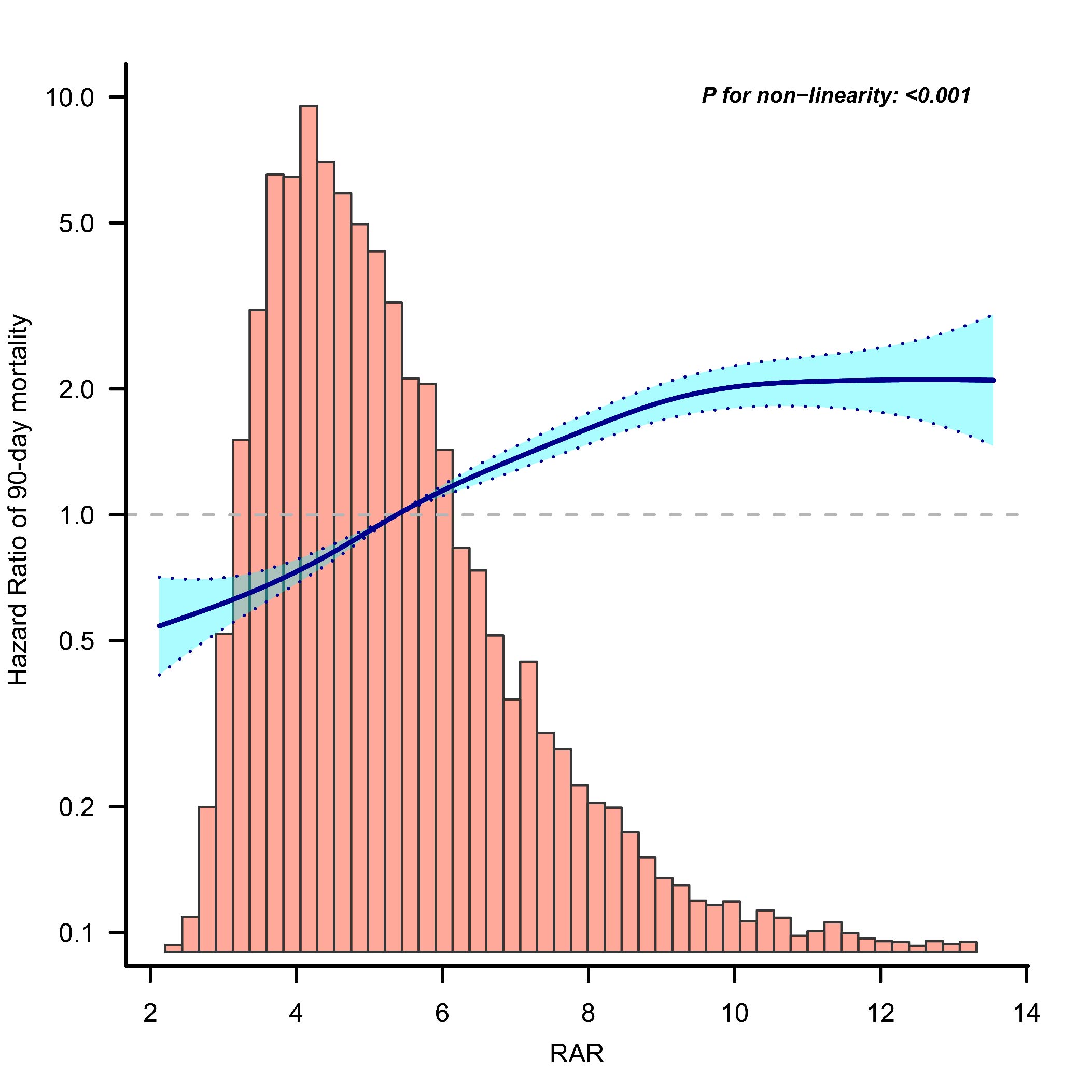

Supplement: Supplementary file 2 [file Image_1.JPEG]

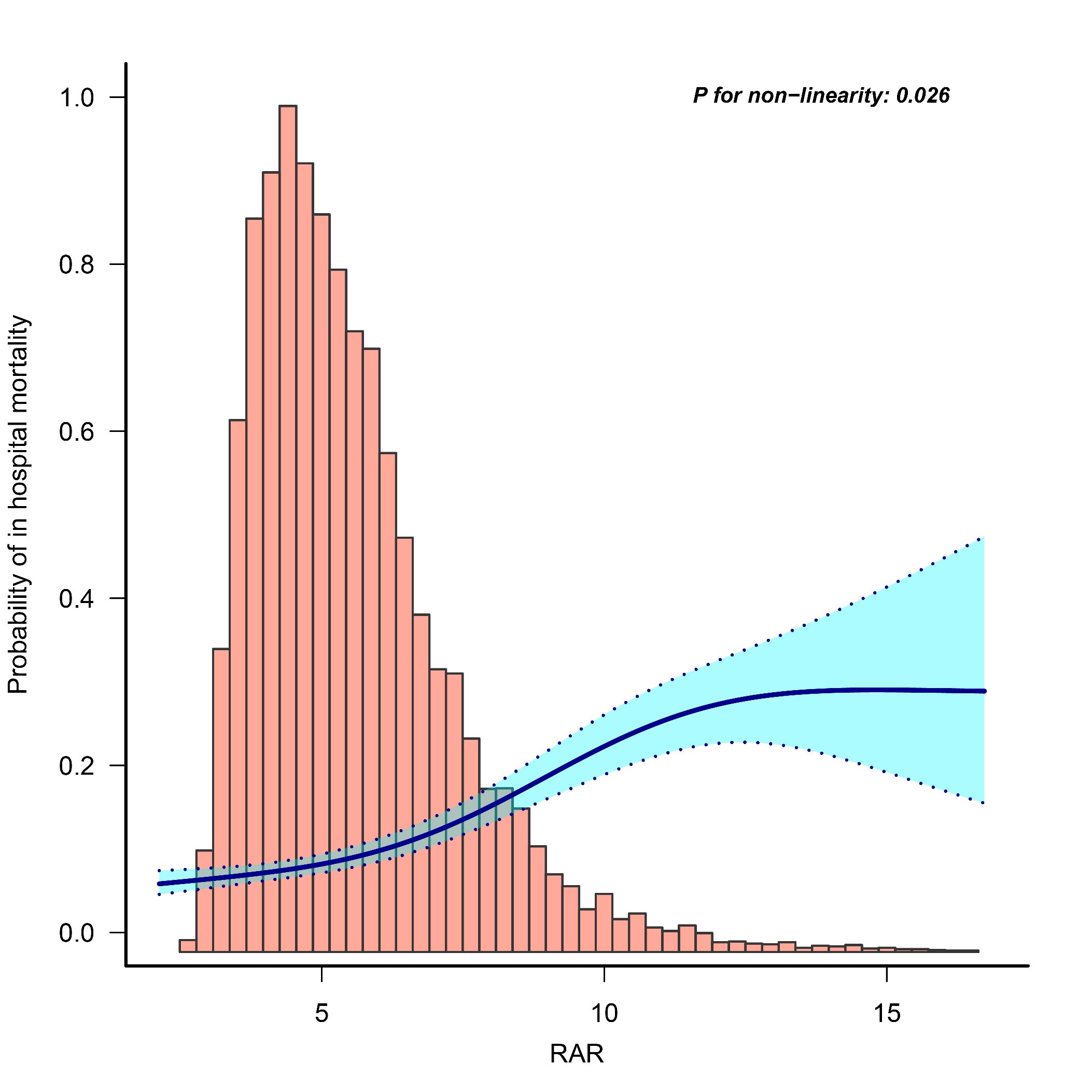

Supplement: Supplementary file 3 [file Image_2.JPEG]

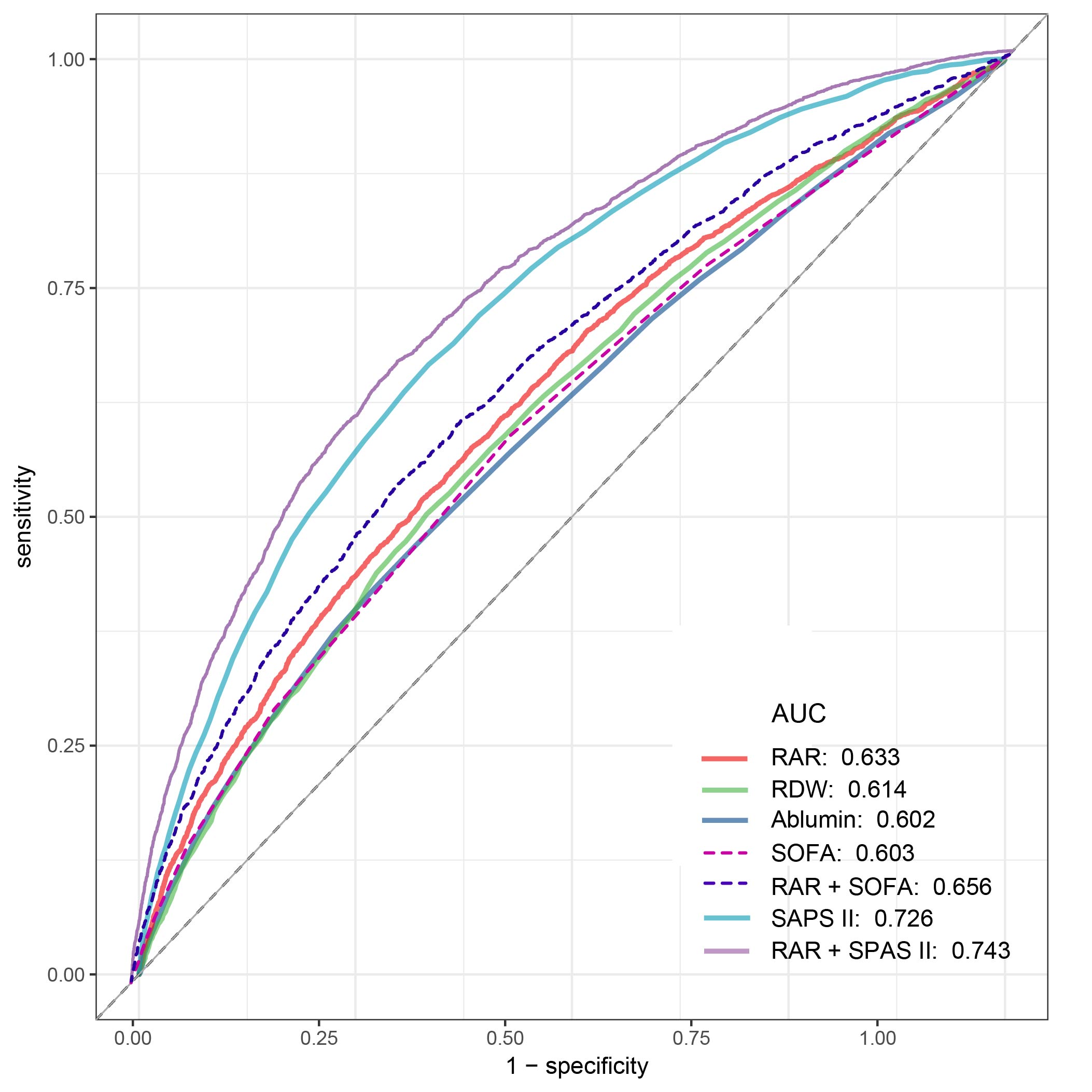

Supplement: Supplementary file 4 [file Image_3.JPEG]
